# Application of spatio-temporal image correlation technology in the diagnosis of fetal cardiac abnormalities

**DOI:** 10.3892/etm.2013.1060

**Published:** 2013-04-09

**Authors:** YIHUA HE, JUNLAN WANG, XIAOYAN GU, YE ZHANG, JIANCHENG HAN, XIAOWEI LIU, ZHIAN LI

**Affiliations:** Department of Ultrasound, Beijing Anzhen Hospital, Capital Medical University, Beijing 100029, P.R. China

**Keywords:** fetal cardiac abnormality, spatio-temporal image correlation technology, fetal echocardiography, pathology

## Abstract

Congenital heart disease is the birth defect with the highest incidence in China. Its timely and accurate prenatal diagnosis is critical for appropriate perinatal and postnatal management and salvage treatment. With improvements in the diagnostic capabilities of ultrasound and clinical manipulation techniques, prenatal diagnosis is conducted increasingly early and with greater accuracy. However, the representations of tiny blood vessels and the determination of abnormal spatial structures in the fetal period continue to cause difficulties in prenatal diagnosis. In theory, spatio-temporal image correlation (STIC) technology is able to compensate for the defects of previous traditional two-dimensional (2D) ultrasound and improve the diagnostic accuracy. The aim of the present study was to investigate the clinical application value of STIC technology combined with traditional 2D ultrasound in the diagnosis of fetal cardiac abnormalities. A total of 1,286 fetuses were subjected to sequential echocardiographic examination, during which STIC technology was used to collect heart volume data and carry out image post-processing and off-line analysis. In addition, the prenatal and postnatal echocardiography results were compared with the pathology results following the induced labor of fetuses with cardiac abnormalities. The sensitivity, specificity, misdiagnosis rate and rate of missed diagnosis for the STIC technology in the diagnosis of prenatal fetal cardiac abnormalities were 97.4, 99.6, 0.4 and 2.6%, respectively. The total coincidence rate was 99.2% and the positive and negative predictive values were 97.9 and 99.4%, respectively; the statistics for the consistency check of the STIC technology in the diagnosis of fetal cardiac abnormalities are κ=0.991, P=0.000. STIC technology combined with fetal echocardiography may be used for the definite diagnosis of fetal heart malformations, with high sensitivity and specificity.

## Introduction

The incidence rate of fetal cardiac abnormalities is reported to be 4–10% (1). By clarifying the diagnosis of fetal cardiac abnormalities and improving the domestic use of such a diagnosis, in addition to taking corresponding preventive measures and intervention strategies, the incidence rate of fetal and infant congenital cardiac malformations is likely to be reduced significantly.

The early diagnosis of complex fetal cardiac abnormalities has become a frontier subject in the prenatal diagnosis field (2). Traditional two-dimensional (2D) echocardiography indicates anatomical details clearly; it has become the gold standard in the prenatal diagnosis of fetal cardiac abnormalities (3–5) and remains the main non-invasive diagnostic method used in the screening and diagnosis of fetal cardiac abnormalities (6). The World Society of Ultrasound in Obstetrics and Gynecology proposes that the necessary views for screening fetal hearts include the four chamber view, the left ventricular outflow tract view, the right ventricular outflow tract view and the three vessel-trachea view (7). However, these views would miss the diagnosis of certain fetal cardiac abnormalities, despite the fact that they include the scanning views of the left ventricular outflow tract, the right ventricular outflow tract and the three vessel-trachea on the basis of the reference four-chamber view. Furthermore, fetal echocardiography has the limitations of a long examination time and high technical difficulty.

Spatio-temporal image correlation (STIC) is a newly developed three-dimensional (3D) imaging technology for fetal hearts and arteries. STIC is able to scan the whole fetal heart within a short time and present the 3D ultrasound image in various imaging modes, which may improve the understanding of the anatomical configuration of the fetal heart. STIC technology may improve the diagnosis rate of fetal cardiac abnormalities and shorten the examination time, in addition to compensating for the deficiencies of traditional 2D echocardiography. Therefore, STIC technology has become a viable and effective method for the diagnosis of fetal cardiac abnormalities.

The current study applied STIC technology in the echocardiographic examination of 1,286 fetuses (18–40 weeks) and evaluated the accuracy of the technology in the diagnosis of fetal cardiac abnormalities.

## Subjects and methods

### Subjects

A total of 1,286 fetuses, which were echocardiographically examined in Beijing Anzhen Hospital (Beijing, China) from September 2010 to September 2011, were selected for the present study and the complete follow-up data of 1,080 fetuses were collected. All the pregnant patients were informed of the details and the agreements to take part in this study were secured from these patients and their families. As for the subjects who were given an autopsy following the termination of pregnancy due to serious fetal cardiac abnormality, the autopsy was carried out in the Pathology Department of the hospital once the pregnant patients and their families had signed an autopsy consent document. This study was conducted in accordance with the Declaration of Helsinki and with approval from the Ethics Committee of Beijing Anzhen Hospital, Capital Medical University. Written informed consent was obtained from all participants.

### STIC technology data collection and image post-processing analysis method

The 2D mode, which was able to present the fetal four chamber view or the main artery arch axis view, was shifted into the 3D/4D mode, during which the pregnant patients were told to hold their breath and the STIC function was initiated. Volume data were collected by setting the collecting time as 7.5–15 sec and the scanning angle as 25–40°, according to the number of gestational weeks, following adjustment of the fields of interest which were included in the volume window. The entire volume data collection process followed the as low as reasonably achievable (ALARA) principle, which meant minimizing the volume of data collected to shorten the scanning time (8–12). The volume probe scanned and collected the volume data of the whole heart automatically and stored it on a hard disk for subsequent off-line analysis.

The volume data images collected by applying STIC technology underwent gray scale and contrast adjustment to achieve satisfactory effects. The dynamic three-orthogonal-plane model, surface imaging mode, reverse mode, tomographic ultrasound imaging technology and vitreous body volume imaging technology were used for off-line analysis according to the diagnostic requirements.

### Statistic analysis

SPSS 16.0 statistical software was used for statistical analysis. The 2×2 table of diagnostic test evaluation was used for evaluating the effectiveness and applicability of 2D ultrasound incorporating STIC technology in the diagnosis of fetal cardiac abnormalities in addition to calculating an evaluating indicator of the diagnostic test. The κ test was used to assess the consistency of 2D ultrasound incorporating STIC technology in the diagnosis of fetal cardiac abnormalities.

## Results

### Clinical data of the subjects

Among the 1,286 subjects, 8 cases were twin pregnancies and the rest were singleton pregnancies. The pregnant patients were 17–43 years old (mean, 28.73±4.60 years) and the fetal gestational age was 18–40 weeks (mean, 26.42±3.94 weeks). Complete follow-up data were collected for 1,080 of the 1,286 subjects (84%), and 206 cases (16%) were missing follow-up data (Table I).

### Comparison of 2D ultrasound incorporating STIC technology with the standard diagnosis method in the diagnosis of fetal cardiac abnormalities

For 2D ultrasound incorporating STIC technology, the sensitivity was 97.4%, the specificity was 99.6%, the misdiagnosis rate was 0.4% and the rate of missed diagnosis was 2.6%. Furthermore, the total coincidence rate was 99.2%, the Youden’s index was 97% and the positive and negative predictive values were 97.9 and 99.4%, respectively (Table II).

**Figure 1:**
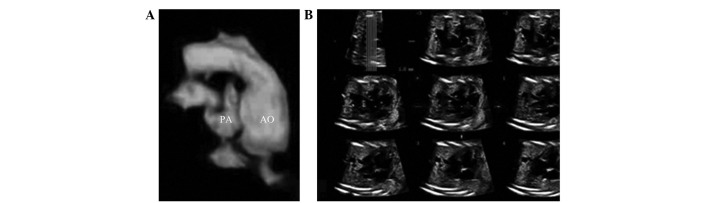
STIC image of the truncus arteriosus communis (Type I) of a 23-week fetus diagnosed by echocardiography. Reconstructed images of (A) the STIC reverse imaging mode and (B) the STIC TUI imaging mode. STIC, spatio-temporal image correlation; TUI, tomographic ultrasound imaging; AO, aorta; PA, pulmonary artery.

**Figure 2:**
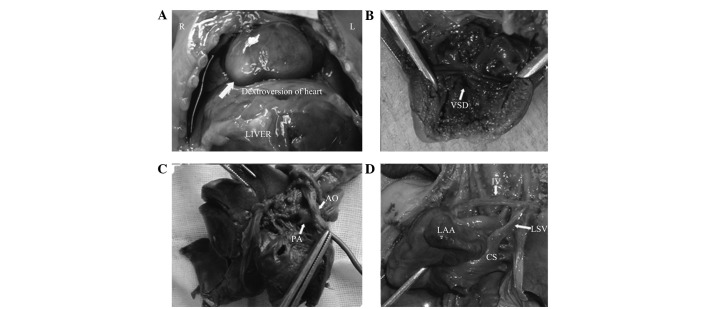
General view of the induced labor autopsy of the case shown in Figure 1. (A) Dextroversion of the heart. (B) A ventricular septal defect. (C) The main pulmonary artery issues from the proximal dorsal aorta. (D) An aneurysm of the left atrial appendage and an expanded coronary sinus that existed in the left superior vena cava. R, right; L, left; VSD, ventricular septal defect; PA, pulmonary artery; AO, aorta; LAA, left atrial appendage; CS, coronary sinus; LSVC, left superior vena cava.

**Figure 3:**
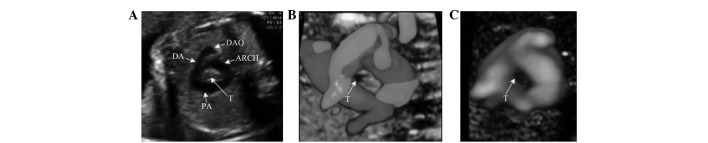
Case of a vascular ring in a 27-week fetus diagnosed by echocardiography. Vascular ring indicated by (A) 2D echocardiography; (B) in vitreous volume imaging indicated by STIC combined with color Doppler; and (C) in volume imaging indicated by STIC combined with power Doppler. T, trachea; PA, pulmonary artery; DA, ductus arteriosus; DAO, descending aorta; ARCH, aortic arch.

**Figure 4:**
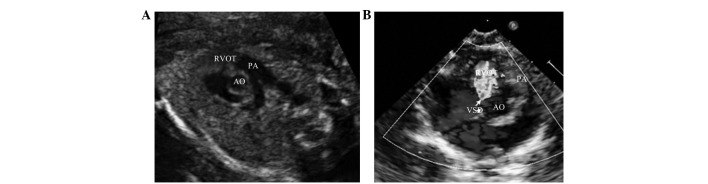
Comparison of a heart diagnosed as normal in a 23-week fetus by echocardiography and as having a ventricular septal defect (membranous part) in a 1-month infant by echocardiography recheck. (A) Fetal echocardiography indicating a normal aorta short axis view. (B) Infant recheck echocardiography revealing a ventricular level shunting signal of the membranous ventricular septum in the color Doppler aorta short axis view. AO, aorta; RVOT, right ventricle of outflow tract; PA, pulmonary artery; VSD, ventricular septal defect.

**Figure 5:**
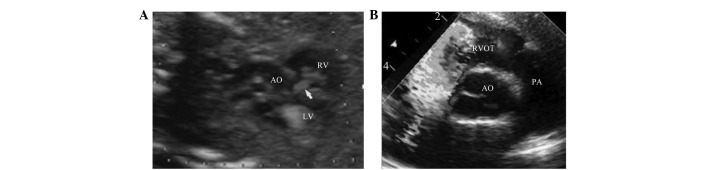
A 25-week fetus was diagnosed with a ventricular septal defect of the membranous part by echocardiography, but a echocardiography recheck in the 42-day infant indicated a normal heart. (A) Fetal echocardiography: color Doppler indicating left to right shunting signal of the left ventricle of the outflow tract (arrow). (B) Infant echocardiography: color Doppler indicating that no ventricular level shunting signal of the membranous ventricular septum was present in the aorta short axis view. AO, aorta; LV, left ventricle; RV, right ventricle; RVOT, right ventricle of outflow tract; PA, pulmonary artery.

The contrasting results of prenatal 2D ultrasound incorporating STIC technology and follow-up testing in false positive and false negative cases of fetal cardiac abnormality are shown in Tables III and IV, respectively. The fetal echocardiography and follow-ups identified 184 true positive cases (17%) (Figs. 1–3), 887 true negative cases (82.1%), 5 false negative cases (0.5%; Fig. 4) and 4 false positive cases (0.4%; Fig. 5).

### Consistency of 2D ultrasound incorporating STIC technology in the diagnosis of fetal cardiac abnormalities

2D ultrasound incorporating STIC technology was compared with the standard diagnosis method in the diagnosis of fetal cardiac abnormalities; the four table diagnostic test evaluation is presented in Table II.

The statistics for the consistency check of the STIC technology were κ=0.991, P=0.000, which indicated that the consistency of the STIC technology was as good as that of the standard diagnostic method.

## Discussion

A prenatal diagnosis is important for the prevention of congenital heart diseases. At present, fetal cardiac ultra-sound is the only effective imaging method for the prenatal diagnosis of cardiac malformation, but misdiagnosed cases continue to occur. Improvements in clinical techniques and the development of new ultrasound technologies for prenatal diagnosis are currently ongoing. STIC is a technology used for fetal echocardiography (13–15), through which complete cardiac information concerning 3D morphology and dynamic changes of the heart structure may be obtained. At the same time, the adjacent location and the spatial relation of the heart structure with the lesion may be displayed. In theory, the 3D heart information is more accessible in the fetal period due to atelectasis. STIC technology may be used as a supplement to traditional ultrasound technology and may replace traditional ultrasound technology to enable the earlier diagnosis of fetal cardiac malformations in the near future (11). STIC technology is a significant method of fetal echocardiography, deserving further study and clinical application. The present study has confirmed the high sensitivity and accuracy of STIC technology combined with traditional 2D ultrasound in the prenatal diagnosis of congenital heart disease.

In this study, the fetal sequential echocardiographic examination method combined with STIC technology was performed on 1,286 fetuses. The results were compared with those of post-natal echocardiography and autopsy following induced labor due to complex cardiac malformation. The STIC technology was identified as useful for displaying the spatial correlation of anatomical structures in fetal cardiac malformations, and is a process that affords high sensitivity and specificity as follows: sensitivity, 97.4%; specificity, 99.6%; positive predictive value, 97.9%; and negative predictive value, 99.4%. In addition, STIC technology also has a high consistency (κ=0.991) for the diagnosis of complex fetal cardiac malformation.

In the cases with the application of STIC technology combined with 2D ultrasound, the complex congenital heart malformation includes infracardiac type pulmonary venous drainage, type III common arterial trunk, coronary circulation dependent right ventricular dysplasia and coronary artery abnormalities. The advantages of STIC technology in the detection of venous drainage terminals, coronary artery origins and tiny blood vessel pathways may complement 2D ultrasound technology and avoid misdiagnosis. Compared with 2D ultrasound, the STIC technology is able to obtain more information with regard to the 3D structure of the heart by reconstruction and superposition. The anatomical structure and adjacent correlations and the lesion range may be observed by graph rotation, translation and cutting. Therefore, the application of STIC technology contributes to the diagnosis of complex fetal heart malformations.

In the present study, 5 cases of false negative heart malformations were identified by comparison of prenatal echocardiography incorporating STIC technology with follow-up data. The misdiagnosed heart malformations included small ventricular septal defects, foramen primum atrial septal defects and mild pulmonary valve stenosis. The retrieval of stored images and retrospective analysis indicated that the 2D ultrasound manifestation of the small ventricular septal defect was concealed. Due to fetal atelectasis, a high pulmonary artery pressure and the pressure balance of the bilateral ventricles, it is not possible for the cross-valve blood flow signal to be displayed, even if using color Doppler ultrasound, which leads to misdiagnosis (16). The location of the atrial septal defect of the primary septum is close to the atrio-ventricular valve and coronary sinus outlet. It is easily sheltered by the valve and confused with the coronary sinus outlet. With STIC technology, the 3D image is recut and the atrial septum may be observed without the sheltering effect of the atrio-ventricular valve. The atrial and ventricular septal defects may be identified by multi-slicing with one point of structure confirmation using STIC technology. However, further experience of the post-processing of STIC 3D images is required to further improve the diagnostic accuracy (17,18).

The present study observed 2 misdiagnosed cases with mild pulmonary valve stenosis. It may be concluded by a comparison of prenatal and postnatal echocardiography that, when diagnosing mild pulmonary valve stenosis by fetal echocardiography, the morphological echo, opening and closing activity and pulmonary valve loop size should be carefully observed to detect the color mixing flow signal and high speed blood flow in the Doppler spectrum. It should be noted that, due to a lower blood flow and mild pulmonary valve stenosis during the fetal period, the stenosis sign may be presented. Subsequent to birth, the increased pulmonary blood flow and cross-valve blood flow may manifest as pulmonary stenosis in the ultrasound image. Therefore, certain mild lesions, including small ventricular septal defects and mild pulmonary valve stenosis, may not be detected due to the technical level of the instrument and the special circulatory status during the fetal period. This should be explained to the patient.

In the present study, 4 false positive cases of cardiac abnormality were identified with a ventricular septal defect or suspicious ventricular septal defect in the fetal echocardiogram and follow-up, but no abnormalities in the postnatal echocardiogram. There is a cross connection between normal left and right ventricular outflow tracts. The reasons for the misdiagnosis with ventricular septal defects may be that there is a mixed blood flow signal in the echocardiography, and the blood flow signal from the right ventricular outflow tract is recognized as the shunting signal from right to left in the ventricular septum or perimembranous region. According to the comparison of prenatal and postnatal echocardiography, it may be concluded that the 2D structure of the ventricular septum should be observed carefully to exclude echo loss in the diagnosis of ventricular septal defects. The color flow gain and sensitivity should be adjusted to the optimal status in the application of color Doppler ultrasound to observe the shunting at the ventricular level. This is likely to improve the accuracy of the prenatal ultrasound and reduce the false positive rate.

The misdiagnosed patients included 2 cases of pulmonary vein atresia and 1 case of left pulmonary hypoplasia. The main cause of this misdiagnosis was the lack of knowledge concerning these two diseases in the fetal period. Atresia with a common arterial trunk is a rare congenital heart disease and is the most serious type of ectopic pulmonary venous drainage. The newborn rarely survives. There has been no experience accumulated in the detection of Atresia by neonatal and adult echocardiography, which thus leads to misdiagnosis. In the present study, the cases with lung agenesis were prenatally diagnosed with partial pulmonary venous ectopic drainage combined with extracardiac malformation, and right pulmonary agenesis combined with other heart defects. The autopsy showed no abnormal cardiac structure. A lack of awareness concerning the effects of extracardiac malformation on blood flow abnormalities is the main cause of misdiagnosis. For cases without a display of blood flow from the right pulmonary vein to the left atrium, the main cause is likely to be pulmonary hypoplasia or an ectopic connection of the pulmonary vein. Therefore, the mastering of heart disease-related knowledge and correct and comprehensive clinical thinking are the basis of improvements in the prenatal diagnosis levels of fetal cardiac diseases.

The present study concluded that STIC technology combined with fetal echocardiography may be used for the definite diagnosis of fetal heart malformations, with high sensitivity and specificity.

## Figures and Tables

**Table I:** Clinical data.

	Age at pregnancy, years			Fetal gestational age, weeks				
	
Cases, n	Range	Mean	SD	Range	Mean	SD	Follow-up, cases	Follow-up, %
1286	17–43	28.73	4.60	19–37	26.42	3.94	1080	84.0

SD, standard deviation.

**Table II:** Comparison of 2D ultrasound incorporating STIC technology with postpartum recheck and autopsy following induced labor in the diagnosis of fetal cardiac abnormalities.

2D ultrasound incorporating STIC technology	Postpartum recheck and induced labor autopsy		

	Positive	Negative	Total
Positive	184	4	188
Negative	5	887	892
Total	189	891	1080

2D, 2-dimensional; STIC, spatio-temporal image correlation.

**Table III:** Comparison of 2D ultrasound incorporating STIC technology in the diagnosis of false positive cases of fetal cardiac abnormality with postpartum recheck and autopsy following induced labor.

Order	2D incorporating STIC technology	Postpartum recheck and induced labor autopsy
1	Excluded ventricular septal defect	Normal
2	Suspicion of ventricular septal defect	Normal
3	Ventricular septal defect (muscular part)	Normal
4	Ventricular septal defect (muscular part)	Normal

2D, 2-dimensional; STIC, spatio-temporal image correlation.

**Table IV:** Comparison of 2D ultrasound incorporating STIC technology in the diagnosis of false negative cases of fetal cardiac abnormality with postpartum recheck and autopsy following induced labor.

Order	2D incorporating STIC technology	Postpartum recheck and induced labor autopsy
1	Normal	Ventricular septal defect and membranous aneurysm formation, patent foramen ovale, tricuspid regurgitation
2	Left ventricular glare point	Pulmonary stenosis (mild), atrial septal defect (II hole, central)
3	Left ventricular glare point	Pulmonary stenosis (mild), patent foramen ovale
4	Normal	Atrial septal defect (II hole, central)
5	Normal	Ventricular septal defect (membranous part)

2D, 2-dimensional; STIC, spatio-temporal image correlation.
